# Maisotsenko Cycle for Heat Recovery in Gas Turbines: A Fundamental Thermodynamic Assessment

**DOI:** 10.1002/gch2.202300178

**Published:** 2023-10-15

**Authors:** Rasikh Tariq, Hakan Caliskan, Nadeem Ahmed Sheikh

**Affiliations:** ^1^ Tecnologico de Monterrey, Institute for the Future of Education, Ave. Eugenio Garza Sada 2501 Monterrey N.L. 64849 Mexico; ^2^ Department of Mechanical Engineering Faculty of Engineering and Natural Science Usak University Usak 64200 Turkey; ^3^ Department of Mechanical Engineering Faculty of Engineering and Technology International Islamic University Islamabad 44000 Pakistan

**Keywords:** educational innovation, exergy analysis, gas turbines, maisotsenko cycle, waste heat recovery

## Abstract

This paper reports the Maisotsenko's cycle‐based waste heat recovery system with enhanced humidification to exploit the maximum waste heat recovery potential of the gas turbine. This research uses an integrated methodology coupling thermodynamic balances with heat transfer model of air saturator. The performance of the system is deduced which are assisted with sensitivity analysis indicating the optimal mass flow rate ratio (0.7–0.8) and pressure ratio (4.5–5.0) between the topping and bottoming cycles, and the air saturator split (extraction) ratio (0.5). The net‐work output, energy, and exergy efficiencies of the system are found to be ≈58.39 MW, ≈55.85%, and ≈52.79%, respectively. The maximum exergy destruction ratios are found as 68.2% for the combustion chamber, 16.0% for the topping turbine, 5.7% for topping compressor, 4.9% air saturator. The integration of Maisotsenko's cycle‐based waste heat recovery system with a comprehensive thermodynamic model, as demonstrated in this research, offers valuable insights into enhancing the efficiency, cost‐effectiveness, and environmental impact of gas turbines. By presenting fundamental equations related to thermodynamic balances, this work serves as an invaluable educational resource, equipping future researchers and students with the knowledge and skills needed to advance the study of thermodynamics and sustainable energy solutions.

## Graphical abstract

This paper reports the selection of optimal parameters and assessments for Innovative waste heat recovery systems using gas turbines for improved energy and exergy efficiency. The innovative systems utilize Maisotsenko's cycle‐based waste heat recovery system with enhanced humidification to exploit the maximum waste heat recovery potential of the gas turbine. Existing research has focused on the usage of holistic and lumped approaches to model Maisotsenko humid air saturator, however, this research uses an integrated methodology coupling thermodynamic balances with heat transfer model of air saturator, thus, this work provides a depth of the coupling of air saturator with the overall thermodynamic model.

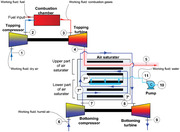

## 1 Introduction

Energy‐efficient conversion and utilization have key importance in the global sustainability goals of the United Nations^[^ ^1^ ^]^ in which the energy production units, i.e., power plants are the pivot points and the purpose is to make them operate‐able with minimum possible resources and maximum benefits, i.e., work. Many methods can be adapted for energy efficiency in the power plants^[^ ^2^ ^]^ ranging from the alteration in the design and/or applying operational health monitoring programs.^[^ ^3^ ^]^ Though, various power plants are inherited with some flaws owing to the nature of the design. For example, the gas turbine power plants are favorable owing to their shorter startup duration, rapid planning, installation, and among many other benefits; nevertheless, integrating them with another air‐based waste heat recovery; in which working fluid air has poor thermophysical characteristics (low thermal conductivity and enthalpy), is not the best alternative to accept the waste heat of a gas turbine topping cycle.

Other waste heat recovery options of topping cycle are to integrate them with organic Rankine cycle,^[^ ^4^ ^]^ CO_2_ cycles,^[^ ^5^ ^]^ humid air cycles,^[^ ^6^ ^]^ extracting other polygeneration processes, integrating an inlet air cooling system^[^ ^7^ ^]^ or a solar farm,^[^ ^8^ ^]^ or through steam injection.^[^ ^9^ ^]^ A rationale to use the humid air bottoming cycle as a waste heat recovery alternative in a gas turbine topping cycle is that the humid air possesses more enthalpy than the dry air and it can increase the net‐work for such a combined cycle. Additionally, the humid air bottoming cycles (HABC) can be implemented in existing air bottoming cycles without any need for redesigning and manufacturing the turbomachinery, making HABC favorable in terms of its operation. In fact, Carrero et al.^[^ ^10^ ^]^ demonstrated that humidification can improve the gas turbine cycle, and various benefits of humid air cycles are summarized by Jonsson and Yan^[^ ^11^ ^]^ mentioning the potential of high electricity efficiency, specific power output, decreased formation of nitrogen oxides in the combustor,^[^ ^12^ ^]^ improved part‐load performance compared to combined cycles, and reduced power output degradation owing to low ambient pressures or high ambient temperatures.

A versatile cycle humidification techniques for the gas turbines are reported in the literature which is categorized by Jonsson and Yan^[^ ^11^ ^]^ into the following: a) injecting steam,^[^ ^13^, ^14^, ^15^ ^]^ b) injecting liquid water in a saturation tower with a water recovery loop, and c) injecting liquid water that will fully evaporate.^[^ ^16^ ^]^ Back in history, Rao^[^ ^17^ ^]^ first patented the humid air turbine cycle using a saturation tower and a water recovery loop, and afterward, many variants including a) cascaded humidified advanced turbines,^[^ ^18^ ^]^ b) regenerative evaporative cycle removing the need of a saturation tower while maintaining similar performance to humid air turbine cycle,^[^ ^19^ ^]^ are available. Technical research on the experimental characterization of humid air cycle using thermodynamic assessment was published by Carrero et al.^[^ ^20^ ^]^ and reported an overall increment of 4.2% absolute points in the electrical efficiency. Wei et al.^[^ ^21^ ^]^ experimentally analyzed the off‐design performance of a small size humid air turbine cycle and reported that the highest efficiency can be increased by 3.1%. Wang et al.^[^ ^22^ ^]^ experimentally analyzed the humidification performance of a spray saturator in a humid air turbine cycle. Orts‐Gonzalez et al.^[^ ^23^ ^]^ demonstrated the economic viability of a reheated humid air turbine concluding a 2.2% lower cost of electricity than simple humid turbine cycles. Xu et al.^[^ ^24^ ^]^ also experimentally evaluated a micro humid air turbine of 100 kW reporting an improvement of ≈22% in electrical efficiency. In another work, Xu et al.^[^ ^25^ ^]^ presented a compact packing humidifier for the micro humid air turbine cycle. Pashchenko et al.^[^ ^26^ ^]^ focused that the thermodynamic efficiency also depends upon the temperature, pressure, and steam‐to‐methane ratio for a chemically recuperated gas turbine and found that the best heat recuperation rate is up to 0.63 for a high temperature gas turbine while using a steam‐to‐methane ratio of 3. Aygun et al.^[^ ^27^ ^]^ conducted an assessment based upon exergy analysis of variable cycle aero‐engine working in its off design conditions, and also presented the thermodynamic, environmental, and sustainability caulcations for a conceptual turboshaft engine.^[^ ^28^ ^]^ On the other side, Pashchenko et al.^[^ ^29^ ^]^ conducted a thermodynamic cycle of an ammonia‐fired chemically recuperated gas turbine. While other research in the application of statistical analysis for the optimal performance of gas turbine power plant by Ibrahim et al.^[^ ^30^ ^]^ and the implementation of regenerative, reheat, intercooler, and complex cycle gas turbine also remained active in research.^[^ ^31^ ^]^

The prime component in a humid air turbine cycle is the efficient design of the humidifier or air saturator where the objective is to maximize the air humidification with the minimum energy consumption. The water management from a purified water body, the injection into the air saturator, and its rejection into the environment from the outlet of the bottoming turbine is an engineering challenge. Paepe et al.^[^ ^32^ ^]^ provided a technical note on water injection‐related issues. In an experimental configuration of Turbec T100 mGT which operates at a constant electric power output condition, the mGT controller algorithm decreases the compressor mass flow rate through a reduction in the shaft speed to keep a constant power production. Water injection happens between the compressor and the turbine which causes an unbalance in the mass flow rate and causes an excess produced electrical power. Here, the controller plays a role, decreasing the shaft speed to maintain constant output electricity, and here, the reduction in the shaft speed can shift the compressor operation to the surge limit. Likewise, some experimentation conducted on steam injection gas turbines (Macchi et al.^[^ ^33^ ^]^) and humid air turbine cycles (Agren et al.^[^ ^34^ ^]^) has shown that condensed water can have environmental consequences and can cause material failure because of its acidic nature, i.e., presence of dissolved CO_2_. Thus, it is an engineering challenge to run a smooth experimental operation of humid air turbines and it is also necessary to add a water treatment procedure before the pumping operation. Nevertheless, one of the requirements is to attain a maximum air humidification rate with a minimum area requirement of the air saturator in the humid air turbines. Therefore, an engineered solution is required for the optimal design of the air saturator with optimal water injection. One of the modifications in the humid air cycle was first proposed by Gillan and Maisotsenko^[^ ^35^ ^]^ which consisted of a unique air saturator having a) an improved indirect evaporative cooler with sub‐wet bulb cooling potential and a b) heat recovery unit. Omar et al.^[^ ^36^ ^]^ provided a comparative study between the Maisotsenko combined cycle (MCC) and the conventional combined (Brayton and Rankine) cycle (CC) and reported that MCC has a higher efficiency than the conventional CC by 6%. Zhu et al.^[^ ^37^ ^]^ carried out a comparative study on humid gas turbines with various configurations of air saturators and reported that the Maisotsenko (M) cycle‐based configuration is quite suitable for humid air turbines. Saghafifar et al.^[^ ^38^ ^]^ also demonstrated that the usage of Maisotsenko cycle‐based air saturators can yield a 7% higher efficiency and ≈44% of work augmentation.

**Figure 1:**
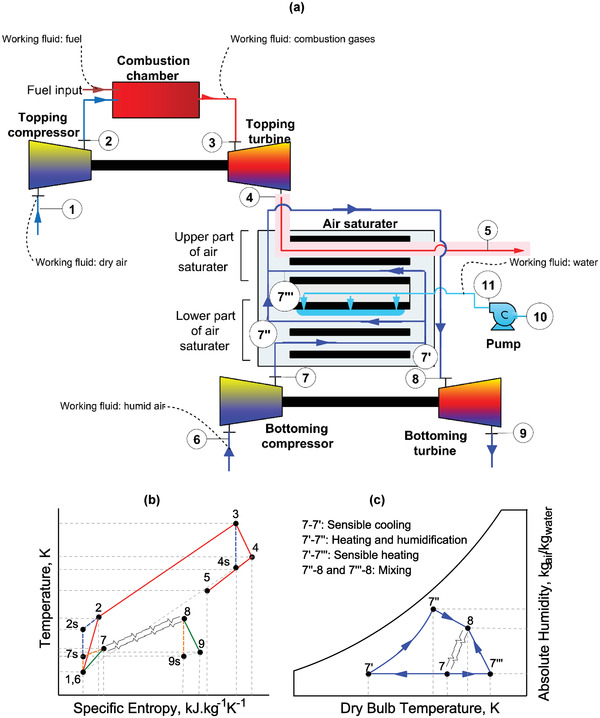
a) Schematic layout of the Maisotsenko humid air bottoming cycle with a gas turbine topping cycle b) T‐s diagram, and c) psychometric representation of the air saturator.

The Maisotsenko cycle‐based air saturator has several geometric and operational complexities and the current objective is to reduce those complexities^[^ ^39^ ^]^ without the cost of energy efficiency. Conventionally, the configuration considers water injection in both parts of the air saturator.^[^ ^35^ ^]^ Later on, Tariq et al.^[^ ^40^ ^]^ has demonstrated that a well‐designed controlled water injection in just the lower part of the air saturator (see **Figure** **1**) can also produce similar energy yield as compared to the configurations having water injections in both parts of the air saturator. Thus, water injection was removed in the upper part of the air saturator while retaining the system performance indicators. It would generate a practically favorable configuration because the high‐pressure complex system of water injection has been decreased to only one, as compared to the previous two injection systems.

Previously, heat transfer analysis is carried out on this configuration of the air saturator to find the optimal air‐vapor mix at the inlet of the bottoming turbine. However, the characterization of the Maisotsenko humid air turbine cycle can be improved by the application of thermodynamic analysis specifically exergy analysis which aids the information of useful energy (availability) part. Such an analysis can help to evaluate the exergetic losses/destructions identifying new ways to increase the system performance. It can be noted from **Table** **1** that previous literature on this area has focused on the energy analysis on a component scale. In this way, various holistic parameters like air saturator degree of humidification, wet‐bulb/dew‐point effectiveness are introduced to acquire additional balance equations for the air saturator, nevertheless, these parameters are the function of operating, geometric, and flow conditions of the air saturator. In another perspective, there is still a lack of extensive exergetic evaluation of such configuration. Although, Paepe et al.^[^ ^41^ ^]^ carried out an exergetic evaluation of one of the configurations of the Maisotsenko humid air turbine cycle (different from the current configuration), nevertheless, the authors of reference^[^ ^41^ ^]^ do not consider the detailed heat transfer based air saturator model and used alternative holistic parameters like dew‐point and wet‐bulb effectiveness to solve the air saturator energy balance. Therefore, the prime novelty of this work is to evaluate the potential of the gas turbine topping cycle with Maisotsenko humid air bottoming cycle using exergy analysis coupled with a detailed heat transfer model of the air saturator. The work now provides a comprehensive and state‐of‐the‐art collection of couple analysis and associated energy and exergetic computations.

**Table 1:** Identification of Gaps with the Research Available in the Literature.

Reference	Year^a)^	Summary	Configuration	Coupled thermodynamic assessment with heat transfer analysis of the air saturator?	Carried out energy analysis?	Carried out exergy analysis?
[35]	2003	This is a conceptual article in which the authors reported the working principle along with the details of the components of the Maisotsenko gas turbine cycle.	Conventional Maisotsenko air cooler	No	No	No
[42]	2011	The authors analyzed the Maisotsenko power cycle for turbine engines through humidifying air recuperator.	Turbine engine coupled with Maisotsenko air humidifier	No	Yes	No
[43]	2015	Two different Maisotsenko thermal machines are considered the first one at the inlet of the compressor, and the second one at the inlet of the combustion chamber. Exergy analysis is presented.	Maisotsenko air cooler is installed in the topping cycle	No	Yes	Yes
[38]	2015	A thermodynamic model of the Maisotsenko gas turbine cycle is developed, and sensitivity analysis is performed to understand the system performance.	Double air saturator based on Maisotsenko cycle	No	Yes	No
[44]	2015	The conventional Maisotsenko gas turbine cycle is analyzed thermodynamically, and a sensitivity analysis is presented.	Double air saturator based on Maisotsenko cycle	No	Yes	No
[36]	2016	A thermodynamic and economic analysis is carried out by placing the Maisotsenko machines in three different locations (inlet of compressor, lower and upper air saturator) of the combined cycle plant.	Double air saturator based on Maisotsenko cycle	No	Yes	No
[45]	2017	A triplex air saturator is employed in the waste heat bottoming cycle, and thermodynamic optimization is carried out to study the merits and demerits of the Maisotsenko bottoming cycle and regenerative Maisotsenko bottoming cycle.	Double and triplex air saturator based on Maisotsenko cycle	No	Yes	No
[46]	2017	The authors integrated a heliostat field collector on the topping cycle of a gas turbine, whereas, the Maisotsenko cycle‐based humid air cycle was used as a waste heat recovery unit.	Double air saturator based on Maisotsenko cycle	No	Yes	No
[47]	2017	Different optimization scenarios are considered for a gas turbine having a Maisotsenko cooler at the inlet of the compressor.	Air saturator based on Maisotsenko cycle for pre‐cooling of the air	No	Yes	No
[40]	2018	The focus of this work is on the numerical heat transfer modeling of the air saturator and an optimized air‐water mixture is calculated on the bottoming air saturator.	Air saturator based on Maisotsenko cycle installed on the waste heat recovery unit	Yes	Yes	No
[39]	2018	The configuration uses a mixed flow air saturator in the bottoming cycle and thermodynamic balance	Mixed flow saturator based on Maisotsenko cycle installed on the waste heat recovery unit	Yes	Yes	No
[48]	2018	In this study, the authors presented a combined cooling, heating, and power system which is driven by a Maisotsenko humid air turbine cycle for a building application.	Combined cooling, heating, and power system are driven by a Maisotsenko Brayton cycle	No	Yes	No
[49]	2019	An absorption chamber and a Maisotsenko air cooler are installed before the compressor of the gas turbine cycle.	Air cooler based on Maisotsenko cycle for pre‐cooling of the air	No	Yes	No
[37]	2019	Three air saturator types (conventional, indirect evaporative cooler, and Maisotsenko cooler) are considered in the topping cycle of the gas turbine, energy analysis is considered, and the air saturator model is solved through control volume formulation.	Single and double Maisotsenko cycle air saturator installed on the topping cycle	Yes	Yes	No
[50]	2019	The authors reported the performance of two different types of humid air gas turbines integrated with a Maisotsenko air saturator.	Humid air gas turbine with Maisotsenko cycle	No	Yes	No
[41]	2020	In this article, the authors have coupled an M‐power cycle to a 100 kW Brayton cycle to carry out the analysis on ASPEN.	M‐power cycle applied to the topping cycle gas turbine.	No	Yes	Yes
[51]	2020	The authors carried out the analysis on an open Maisotsenko Brayton cycle using finite‐time thermodynamics theory and reported that such a type of configuration has better thermal performance as compared to the conventional Brayton cycles.	Open Maisotsenko‐Brayton cycle	No	Yes	No
This work	2023	Energy and exergy analyses on the combined cycle power plant having Maisotsenko air saturator at the bottoming cycle are presented. The work combines the heat and mass transfer dimensions of the air saturator, consisting of the control volume mathematical model, with the exergetic analysis evaluating entropy generation rate, and relative exergy destruction.	Modified and more practical Maisotsenko air saturator installed in the waste heat recovery unit	Yes	Yes	Yes

^a)^ Literature is sorted based upon chronological order.

Another contribution of this work is to present the sensitivity of the split ratio of dry airflow and the wet airflow inside the air saturator which is normally considered 0.3 as an input parameter and not considered as a tuning parameter.^[^ ^38^ ^]^ This split ratio has high importance, because, it governs the role of upper and lower air saturators in the bottoming cycle, and any improper selection of this parameter can drive the system to operate at a lower efficiency point owing to the non‐desired high water injection rates which can be crucial.

In this work, global mass, energy, and exergy balance models are developed for Maisotsenko humid air turbine cycle for gas turbine bottoming cycle. The balance models are assisted with the heat transfer model previously published by the authors.^[^ ^40^ ^]^ The performance is gauged using the overall work output, first law efficiency, second law efficiency, relative exergy destruction, and entropy generation rate.

## 2 System Description

The schematic layout of the Maisotsenko humid air bottoming cycle with a gas turbine topping cycle is shown in Figure 1a. Ambient air at state 1 enters the topping compressor. It is compressed from state 1 to state 2 reaching a relatively high temperature and pressure depending upon the pressure ratio of the compressor (see Figure 1b). The compressed air enters the combustion chamber where the fuel is injected through an appropriate air‐fuel ratio. The combustion process is exothermic in which heat is released. This heat is captured in the form of combustion gases and leaves the combustion chamber at state 3. The combustion chamber at high temperature and pressure are suitable enough to run the turbine with the objective to generate work. At this point, the combustion gases leave the topping cycle and enter the bottoming cycle.

In the bottoming cycle, ambient air at state 6 enters the bottoming compressor where it is compressed to a state 7. This compressed air enters the bottoming part of the air saturator. The objective of the bottoming part of the air saturator is to humidify the air. This process has vital importance in the whole power plant because humid air has high enthalpy as compared to dry air. Therefore, appropriate air humidification is necessary to fully extract the heat in the form of waste heat recovery. The lower section has a wet and dry channel, and a pump is used to inject the water inside the air saturator at state 11. The compressed air enters the dry channel of the air saturator and it is cooled to sub‐wet‐bulb temperature reaching at state 7′ through a sensible cooling (see Figure 1c) theoretically below its wet‐bulb temperature. Here, it is divided into two parts. The first part enters the wet channel of the lower saturator and it is humidified and slightly heated reaching a state 7′'. It is slightly heated to cool down the temperature of the dry air. The other part enters the upper part of the air saturator where it is heated up to a state 7′“” through contact with the exhaust gases of the gas turbine which finally leaves the system at state 5. Here, the humid air from the lower saturator and the hot air from the upper saturator is mixed up reaching a state 8. Here, it should be noted that the humidity of the air from state 7′ to 7′’’ does not increase because the upper part of the air saturator does not have any water supply, therefore, the current configuration is different from the originally proposed Maisotsenko humid air cycle.^[^ ^35^ ^]^ It is also pertinent to mention that the exhaust gases from the topping cycle do not physically mix with the bottoming air working fluids, and their interaction is heat transfer only. In summary, the lower part of the air saturator serves for humidification purposes, whereas, the upper part of the air saturator is the driving agent of the waste heat recovery unit because it accepts the heat from the topping cycle. Finally, the mixture is expanded into the bottoming turbine generating the work of the bottoming turbine.

It can be noted that the upper part of the air saturator does not have any air humidification. Therefore, this configuration is more practical as compared to the one reported in the literature^[^ ^38^, ^39^, ^44^, ^46^, ^52^ ^]^ because the controlled, optimal, and appropriate humidification at the lower part of the air saturator is fully extracted the waste heat from the topping cycle.

## 3 Modeling Setup

The modeling setup consists of mass, energy, and exergy balances which are applied on each component considering a set of assumptions including a) state‐state operation of all the components,^[^ ^53^ ^]^ and b) the kinetic and the potential energy terms are ignored.^[^ ^54^ ^]^ Apart from the aforementioned assumptions, the combustion chamber has the following assumptions: c) the combustion gases and air are ideal gas mixtures,^[^ ^55^ ^]^ e) 2% of the exhaust combustion gases are lost,^[^ ^56^ ^]^ and f) throttling losses and fuel supply pressure are ignored. Assumption f) can be justified through the selection of the thermodynamic system and in this case, it does not include the fuel injection system. In the case of air saturator, apart from the assumption (a) and (b), the following are valid, g) the boundary walls of the air saturator are adiabatic, h) the developed mathematical model is one dimensional solved in the airflow direction, i) radiative heat flux from the exhaust gases is negligible, j) the thermophysical properties of the air changes along with the flow direction but they remain constant in any given cross‐section,^[^ ^57^ ^]^ k) hydrodynamic, thermal and concentration entry lengths are neglected,^[^ ^58^ ^]^ l) Reynolds analogy holds and the Lewis number is unity,^[^ ^59^ ^]^ and m) the water film immediately next to the airflow is fully saturated and offers negligible thermal and mass transfer resistance at the air‐water interface.

### 3.1 Mass, Energy, and Exergy Balances

The mass balance of the system is given as follows:

(1) $$\sum_{\mathrm{in}}{\dot{m}}_{\mathrm{in}}=\sum_{\mathrm{out}}{\dot{m}}_{\mathrm{out}}$$

The general energy balance on the system is given by the following equation while ignoring the kinetic and potential energy terms:

(2) $${\dot{Q}}_{\mathrm{net},\mathrm{in}}+\sum_{\mathrm{in}}\dot{E}n_{\mathrm{in}}={\dot{W}}_{\mathrm{net},\mathrm{out}}+\sum_{\mathrm{out}}\dot{E}n_{\mathrm{out}}$$

The general expression for the exergy balance is given by the following equation:

(3) $$\dot{E}x_{xd}=\sum_{\mathrm{in}}\dot{E}x_{\mathrm{in}}-\sum_{\mathrm{out}}\dot{E}x_{\mathrm{out}}-\sum \dot{E}x_{\mathrm{loss}}$$

With

(4) $$\dot{E}n=\dot{m}h$$

(5) $$\dot{E}x=\dot{m}\dot{e}x$$

In Equation (3), the term $\dot{E}x_{\mathrm{in}}$ and $\dot{E}x_{\mathrm{out}}$ refers to the flow exergy at the inlet and the outlet of the system. The term $\dot{E}x_{loss}$ is visualized as external irreversibility which is the transfer of exergy from the system to the surroundings possibly, one reason can be heat transfer from the system to the surrounding. The term $\dot{E}x_{xd}$ is an internal phenomenon of the system which refers to the exergy destroyed because of the irreversibilities within the component or system.

The reference conditions involve the value of the temperature, pressure, and relative humidity which are 25 °C, 101.325 kPa, and 60% (corresponding to an absolute humidity of 0.012 kg_vapor_/kg_dryair_, and 0.019 of mole fraction), respectively.

#### 3.1.1 Topping Compressor

The mass balance:

(6) $${\dot{m}}_{1}={\dot{m}}_{2}$$

The energy balance:

(7) $${\dot{W}}_{TC}=\dot{E}n_{2}-\dot{E}n_{1}$$

Equation (7) gives the theoretical rate of work of the compressor, and it is multiplied by $\frac{\eta_{\mathrm{generator}}}{\eta_{\mathrm{mechanical}}}$as a correction factor. The generator and mechanical efficiencies are 98% and 99%, respectively.^[^ ^44^ ^]^

The exergy balance:

(8) $$\dot{E}x_{xd,TC}={\dot{W}}_{TC}+\dot{E}x_{1}-\dot{E}x_{2}$$

The outlet temperature of the compressor is using the compressor isentropic efficiency with constant specific heat, and the pressure ratio; among others, and it is given by^[^ ^55^ ^]^:

(9) $$T_{2}=T_{1}+\frac{T_{2s}-T_{1}}{\eta_{s,TC}}$$

where *T*_2*s*_ is evaluated at entropy at state 1 (*s*_1_) and pressure at state 2 (*P*_2_) depending on the pressure ratio across the topping compressor.

#### 3.1.2 Combustion Chamber

The mass balance:

(10) $${\dot{m}}_{2}+{\dot{m}}_{\mathrm{fuel}}={\dot{m}}_{3}$$

The energy balance:

(11) $$\dot{E}n_{2}+\dot{E}n_{\mathrm{fuel}}=\dot{E}n_{3}+\dot{E}n_{\mathrm{losses},\mathrm{CC}}$$

with

(12) $$\dot{E}n_{\mathrm{fuel}}={\dot{m}}_{\mathrm{fuel}}LHV$$

The state point at 3 is a combination of the exhaust gases. Therefore, the enthalpy at this state point is the summation of the individual properties of each gas having different mass flow rates but pertaining to the same temperature point. It is given by:^[^ ^60^ ^]^

(13) $$\dot{E}n_{3}={\left({\dot{m}}_{N_{2}}h_{N_{2}}+{\dot{m}}_{O_{2}}h_{O_{2}}+{\dot{m}}_{{\mathrm{CO}}_{2}}h_{{\mathrm{CO}}_{2}}+{\dot{m}}_{H_{2}O}h_{H_{2}O}\right)}_{T_{3}}$$

The combustion reaction equation is solved to find the mass flow rates of each individual item in the mixed composition of the exhaust gases. To write the combustion reaction, it is necessary to involve all the hydrocarbons in the natural gas, which is the fuel for the gas turbine, and must also involve all the components of the air too. Their description is given as follows:

1. The molar composition of air is 0.7748 for N_2_, 0.2059 for O_2_, 0.0003 for CO_2_, and 0.019 for H_2_O. The molar composition of air depends, particularly on the outdoor environmental characteristics. In fact, it is also subjected to the geographical location and the conditions of the vicinity of the operation of the power plant. The molar composition also changes with the physical time, for example, daily CO_2_ has reached 419.18 ppm on June 20, 2021^[^ ^61^ ^]^ which can alter the molar composition. Nevertheless, for the sake of the applied nature of the problem, this molar composition of air is taken which is also in agreement with the following references: Dincer and Rosen^[^ ^62^ ^]^ (Example 3.4), and Gürtürk and Öztop.^[^ ^63^ ^]^
2. The composition of the natural gas can vary depending on the quality of the available reservoir and the refining process. The molar composition of natural gas is 0.9194 for methane gas, 0.0353 for ethane gas, 0.009 for propane gas, 0.0038 for butane gas, 0.0011 for pentane gas, 0.026 for N_2_, and 0.0048 for CO_2_.^[^ ^64^ ^]^ This molar composition corresponds to vapor pressure of 0.019 atm in the total air mixture where the saturation pressure is 0.03127 atm. This eventually reflects that the relative humidity of the water in the dry air is approximately 60%. The definition of the environmental reference state conditions including temperature, pressure, and relative humidity are important to use the correct model of the specific molar chemical exergy which is discussed in the subsequent sections.

The combustion equation can be written as:

(14) $$\begin{array}{ccc} & & \left(0.9194{\mathrm{CH}}_{4}+0.0353C_{2}H_{6}+0.009C_{3}H_{8}+0.0038C_{4}H_{10}\right.\\ & & \;\left.+0.0011C_{5}H_{12}+0.026N_{2}+0.0048{\mathrm{CO}}_{2}\right)\\ & & \;+\bar{\lambda }\left(0.2059O_{2}+0.7748N_{2}+0.019H_{2}O+0.0003{\mathrm{CO}}_{2}\right)\\ & & \;\to x_{N_{2}}N_{2}+x_{O_{2}}O_{2}+x_{{\mathrm{CO}}_{2}}{\mathrm{CO}}_{2}+x_{H_{2}O}H_{2}O \end{array}$$

where $\bar{\lambda }$ is the molar air/fuel ratio ( = 26.480^[^ ^64^ ^]^). The calculation result has shown that the molar fractions of the exhaust gases after the combustion chamber have the following percentages: 74.66% for N_2_, 12.40% for O_2_, 3.82% for CO_2_, and 9.12% for H_2_O. These findings also comply with the one reported by.^[^ ^64^ ^]^

The exergy balance:

(15) $$\begin{array}{ccc} \dot{E}x_{2} & + & \dot{E}x_{\mathrm{fuel}}=\dot{E}x_{3}+\dot{E}x_{xd,CC}+\dot{E}x_{\mathrm{losses},\mathrm{CC}}\;\mathrm{or}\;\dot{E}x_{xd,CC}=\dot{E}x_{2}\\ & & +\;\dot{E}x_{\mathrm{fuel}}-\dot{E}x_{3}-\dot{E}x_{\mathrm{losses},\mathrm{CC}} \end{array}$$

The exergy losses from the combustion chamber in the form of heat transfer are calculated using:

(16) $$\dot{E}x_{\mathrm{losses},\mathrm{CC}}=\left[1-\frac{T_{0}}{T_{\mathrm{combustion}}}\right]\dot{E}n_{\mathrm{losses},\mathrm{CC}}$$

However, Equation (16) holds provided it is assumed that the temperature profile is constant over the surface of the combustion chamber. Although, this assumption is debatable and cannot be valid, thus, it is challenging to estimate the term $\dot{E}x_{\mathrm{losses},\mathrm{CC}}$. One alternative is to evaluate $\dot{E}x_{xd,CC}+\dot{E}x_{\mathrm{losses},\mathrm{CC}}$ from the exergy balance equation (Equation (15)). Thus, Equation (15) can be written as:

(17) $$\left(\dot{E}x_{xd,CC}+\dot{E}x_{\mathrm{losses},\mathrm{CC}}\right)=\dot{E}x_{2}+\dot{E}x_{\mathrm{fuel}}-\dot{E}x_{3}$$

The chemical exergy of the fuel is:

(18) $$\dot{E}x_{\mathrm{fuel}}={\dot{m}}_{\mathrm{fuel}}\dot{e}x_{\mathrm{fuel}}$$

The specific chemical exergy of the fuel $\dot{e}x_{\mathrm{fuel}}$can be evaluated using an exergy‐to‐energy ratio abbreviated by φ, and it is given by the expression:

(19) $$\dot{e}x_{\mathrm{fuel}}=\varphi LHV$$

Kotas^[^ ^65^ ^]^ conveyed the empirical relations made by Szargut and Styrylska^[^ ^66^ ^]^ and can be used when the composition of the fuel is not known. Here, the composition of the fuel is known exactly, thus, the data from Szargut et al.^[^ ^67^ ^]^ (Tables I and II) can be applied directly which will yield φ = 1.058. Given these details, the chemical exergy of the fuel can be written as:

(20) $$\dot{E}x_{\mathrm{fuel}}={\dot{m}}_{\mathrm{fuel}}\varphi LHV$$

The total exergy at state point 3 is calculated through the specific exergy. However, since, the exhaust gases contain the combustion products, therefore, the specific exergy on the molar basis $\left(\bar{ex}\right)$ at state point 3 is equal to the chemical and physical exergy, given by the equation:

(21) $${\bar{ex}}_{3}={\bar{ex}}_{\mathrm{chemical},3}+{\bar{ex}}_{\mathrm{physical},3}$$

Additionally, there are four different components which are the combustion products, therefore, the molar exergy at state point 3 can be written as:

(22) $$\begin{array}{ccc} \bar{ex_{3,N_{2}}} & = & {\left({\bar{ex}}_{\mathrm{chemical},N_{2}}+{\bar{ex}}_{\mathrm{physical},N_{2}}\right)}_{3};\bar{ex_{3,O_{2}}}\\ & = & {\left({\bar{ex}}_{\mathrm{chemical},O_{2}}+{\bar{ex}}_{\mathrm{physical},O_{2}}\right)}_{3};\\ \bar{ex_{3,CO_{2}}} & = & {\left({\bar{ex}}_{\mathrm{chemical},CO_{2}}+{\bar{ex}}_{\mathrm{physical},CO_{2}}\right)}_{3};\bar{ex_{3,H_{2}O}}\\ & = & {\left({\bar{ex}}_{\mathrm{chemical},H_{2}O}+{\bar{ex}}_{\mathrm{physical},H_{2}O}\right)}_{3} \end{array}$$

The solution of Equation (22) consists of the calculation of the chemical and physical exergies of each component. The following steps should be considered to find these exergies.

1. The Physical Exergy is given by:
(23) $${\bar{ex}}_{\mathrm{physical},i}=\left(\bar{h_{i}}-\bar{h_{0,i}}\right)-T\left(\bar{s_{i}}-\bar{s_{0,i}}\right)$$
2. Kotas^[^ ^65^ ^]^ described the molar chemical exergy of an individual component which is given by:
(24) $${\bar{ex}}_{\mathrm{chemical},i}=\sum y_{i}{\bar{ex}}_{\mathrm{chemical},i}^{k}+\bar{R}T_{0}\sum y_{i}\ln y_{i}$$

In this equation, *y_i_* is the molar fraction of the combustion products which is calculated by dividing the moles of each of the combustion gas with respect to the total number of moles in the exhaust gases. ${\bar{ex}}_{\mathrm{chemical}}^{i}$is the standard molar chemical exergy, and $\bar{R}$ is the universal gas constant. The standard molar chemical exergy $\left({\bar{ex}}_{\mathrm{chemical},i}^{k}\right)$ for the combustion products including oxygen, carbon dioxide, water vapor, and nitrogen gases are 3970, 19870, 9490, and 720 J mol^−1^, respectively. It is to be stressed here that the standard molar chemical exergy of the substances is debatable because different researchers have used different environmental conditions for the establishment. In fact, Gharagheizi et al.^[^ ^68^ ^]^ has considered them to be a key challenge in the computation of the exergy analysis. Moran et al.^[^ ^69^ ^]^ (Appendix A‐26) has summarized two famous models (model I and model II) which are developed in two different atmospheric pressures (1.019 atm and 1.0 atm) and Moran et al.^[^ ^69^ ^]^ have mentioned, “*…..on an overall basis, the chemical composition of the exergy reference environment of Model II is closer than Model I to the composition of the natural environment….” In fact*, Moran et al.^[^ ^69^ ^]^ have mentioned in section 13.7, “*Standard exergy Model II is commonly used today. Model I is provided to show that other standard reference environments can at least be imagined”* and *“Only one of the two models should be used in a particular analysis*”. Therefore, it can be summarized that the standard molar chemical exergy of model II is valid and can be used for application purposes. Model II of standard molar chemical exergy is also used by Dincer and Rosen,^[^ ^62^ ^]^ i.e., example 3.4 uses model II of standard molar chemical exergy and also shows the details of the reference environmental conditions and the same is adapted in this work. Bejan, Tsatsaronis, and Moran^[^ ^70^ ^]^ have also mentioned both of these models. There is another point of discussion for consideration about the standard of the reference environment related to the model of standard molar chemical exergy. Their value also depends upon the relative humidity of the standard environmental conditions. In fact, notable research is also available in this area published by Ivar Ståle Ertesvåg.^[^ ^71^ ^]^ This reference has provided a detailed description of the variations of chemical exergy with ambient temperature, pressure, and relative humidity. The literature has been seen to be agreeing on the standard temperature and pressure (model II, Appendix A‐26, Moran et al.^[^ ^69^ ^]^), thus, here it is necessary to add a description of the standard relative humidity of the environment for the evaluation of standard molar chemical exergy. Ivar Ståle Ertesvåg^[^ ^71^ ^]^ has shown that the relative deviation in chemical exergy for the methane gas was more than 1% within a change of relative humidity from 10% to 100% at a fixed temperature. Similarly, the standard molar chemical exergy has shown a deviation ranging from −1.5% to 2.5% for O_2_, CO_2_, and H_2_ with the change of relative humidity from 10% to 100%. Even though it is agreed that the relative humidity critically changes the chemical exergy of the substances, but Ivar Ståle Ertesvåg^[^ ^71^ ^]^ has also provided a recommendation on the situation when it is not required to use the reference values regardless of the ambient conditions. The author^[^ ^71^ ^]^ has stated, “*If, on the other hand, the purpose is to compare installations at different locations, it may be interesting to evaluate the plants on a generic basis*, e.g.*, output* per *unit of input mass. In that case, the chemical exergy of the substance at reference conditions may be the useful measure*”. Thus, it is understood that for the applied nature of problems such as the one discussed here, it is alright to use standard molar chemical exergy regardless of the ambient conditions. On the other hand, Dincer and Rosen^[^ ^62^ ^]^ have summarized it (narrating exactly), “*To simplify, Szargut* et al*. (1988) suggested that the chemical exergy obtained in the standard state at normal temperature and pressure should be considered as standard chemical exergy with respect to the conventional mean concentrations of the reference species in the environment*.” Moran et al.^[^ ^69^ ^]^ have also discussed this matter by mention in section 13.7, “*The use of a table of standard chemical exergies often simplifies the application of exergy principles. However, the term standard is somewhat misleading, for there is no one specification of the environment that suffices for all applications. Still, chemical exergies calculated relative to alternative specifications of the environment are generally in good agreement. For a broad range of engineering applications, the convenience of using standard values generally outweighs the slight lack of accuracy that might result. In particular, the effect of slight variations in the values of T_0_ and P_0_ about their standard values normally can be neglected.”*

3) It is also desired to calculate the mass flow of each individual gases to find the total flow exergy of the combustion products. It is calculated using the procedure explained here. The mass fraction is calculated by using the mass in kilograms of each individual gas with respect to the total mass in kilograms. In a general format, it can be given as:
(25) $$\mathrm{Mass}\mathrm{fraction}=\frac{m_{i}}{m_{{\mathrm{CO}}_{2}}+m_{H_{2}O}+m_{O_{2}}+m_{N_{2}}}$$

The physical mass in the units of kilogram in Equation (25) is calculated by:

(26) $$m_{i}=x_{i}\times {\mathrm{Molar}\mathrm{mass}}_{i}$$

In Equation (26), *x_i_* (units: kmol) refers to the composition of the individual gas *i* in the exhaust gases and it comes from the balance of combustion equation (Equation (14)). The units of *m_i_* become kg by the multiplication of *x_i_* (units: kmol) and molar mass (units: kg/kmol).

Equation (24) can be solved using Equation (25), (26), and standard molar chemical exergies $\left({\bar{ex}}_{chemical,i}^{k}\right)$ of each exhaust product. In other words, four molar chemical exergies $\left({\bar{ex}}_{chemical,i}\right)$ are computed corresponding to the fraction ratio (*y_i_*) and standard molar chemical exergy $\left({\bar{ex}}_{chemical,i}^{k}\right)$ for N_2_, O_2_, CO_2_, and H_2_O (Equation 24). Similarly, four molar physical exergies $\left({\bar{ex}}_{physical,i}\right)$ are calculated corresponding to N_2_, O_2_, CO_2_, and H_2_O with each having its own $\bar{h_{i}},\bar{h_{0,i}},\bar{s_{i}},\bar{s_{0,i}}$ properties. At this stage, the summation of individual molar chemical exergy $\left({\bar{ex}}_{chemical,i}\right)$ and molar physical exergy $\left({\bar{ex}}_{physical,i}\right)$ is calculated to yield the total molar exergy for each N_2_, O_2_, CO_2_, and H_2_O $\left(\bar{ex_{3,N_{2}}},\bar{ex_{3,O_{2}}},\bar{ex_{3,CO_{2}}},\bar{ex_{3,H_{2}O}}\right)$ as described in Equation. (21) and (22). The units of each individual molar exergies $\bar{ex_{3,N_{2}}},\bar{ex_{3,O_{2}}},\bar{ex_{3,CO_{2}}},\bar{ex_{3,H_{2}O}}$ are J/kmol (converted into kJ/kmol). Here, each individual molar exergy is divided by the molar mass (units: kg/kmol) of each gas to attain four different mass basis specific exergy $\left(ex_{3,N_{2}},ex_{3,O_{2}},ex_{3,CO_{2}},ex_{3,H_{2}O}\right)$ expressed in the units kJ/kg.

Finally, the total exergy at state point 3 can be calculated using:

(27) $$\dot{E}x_{3}={\dot{m}}_{CO_{2}}ex_{3,CO_{2}}+{\dot{m}}_{H_{2}O}ex_{3,H_{2}O}+{\dot{m}}_{O_{2}}ex_{3,O_{2}}+{\dot{m}}_{N_{2}}ex_{3,N_{2}}$$

The mass flow rates (units: kg/s) $\left({\dot{m}}_{CO_{2}},{\dot{m}}_{H_{2}O},{\dot{m}}_{O_{2}},{\dot{m}}_{N_{2}}\right)$ of individual gases appearing in Equation (27) are calculated using the mass fraction and the mass flow rate (kg/s) at state 3 $\left({\dot{m}}_{3}\right)$ which is obtained from Equation (10). It can be written as:

(28) $${\dot{m}}_{i}=y_{i}\times {\dot{m}}_{3}$$

#### 3.1.3 Topping Turbine

The mass balance:

(29) $${\dot{m}}_{3}={\dot{m}}_{4}$$

The energy balance:

(30) $${\dot{W}}_{TT}=\dot{E}n_{3}-\dot{E}n_{4}$$

Work of topping turbine is multiplied with η_*generator*_η_*mechanical*_.

The exergy balance:

(31) $$\dot{E}x_{xd,TT}=\dot{E}x_{3}-\dot{E}x_{4}-{\dot{W}}_{TT}$$

It should be noted that the energy and exergy equations at state 4 are calculated for the individual energy and exergy rates of all the combustion gases including physical and chemical exergy rates as in point 3 in Section 3.1.2.

#### 3.1.4 Bottoming Compressor

The mass balance:

(32) $${\dot{m}}_{6}={\dot{m}}_{7}$$

The energy balance:

(33) $${\dot{W}}_{BC}=\dot{E}n_{7}-\dot{E}n_{6}$$

A correction factor $\frac{\eta_{generator}}{\eta_{mechanical}}$ is also applied to the work of the bottoming compressor.

The exergy balance:

(34) $$\dot{E}x_{xd,BC}={\dot{W}}_{BC}+\dot{E}x_{6}-\dot{E}x_{7}$$

#### 3.1.5 Pump

The mass balance:

(35) $${\dot{m}}_{10}={\dot{m}}_{11}$$

The energy balance:

(36) $${\dot{W}}_{P}=\dot{E}n_{11}-\dot{E}n_{10}$$

The pump work is needed for the humidification and draws on the produced power. Therefore, a correction factor $\frac{\eta_{generator}}{\eta_{mechanical}}$ is multiplied with the theoretical pump work which accommodates the electrical generation mechanical efficiencies.

The exergy balance:

(37) $$\dot{E}x_{xd,P}={\dot{W}}_{P}+\dot{E}x_{10}-\dot{E}x_{11}$$

#### 3.1.6 Air Saturator

The mass balance^[^ ^72^ ^]^ on the air saturator:

(38) $$\left({\dot{m}}_{7,da}+\omega_{7}{\dot{m}}_{7,da}\right)+{\dot{m}}_{4}+{\dot{m}}_{11}=\left({\dot{m}}_{8,da}+\omega_{8}{\dot{m}}_{8,da}\right)+{\dot{m}}_{5}$$

The mass balance on the exhaust steam in the upper part of the air saturator:

(39) $${\dot{m}}_{4}={\dot{m}}_{5}$$

The energy balance on the overall air saturator:

(40) $$\dot{E}n_{7}+\dot{E}n_{4}+\dot{E}n_{11}=\dot{E}n_{5}+\dot{E}n_{8}$$

The exergy balance:

(41) $$\begin{array}{ccc} \dot{E}x_{7} & + & \dot{E}x_{4}+\dot{E}x_{11}=\dot{E}x_{5}+\dot{E}x_{8}+\dot{E}x_{xd,AS}\;\text{or}\;\dot{E}x_{xd,AS}=\dot{E}x_{7}+\dot{E}x_{4}\\ & & +\;\dot{E}x_{11}-\dot{E}x_{5}-\dot{E}x_{8} \end{array}$$

The energy and exergy equations at state 5 are computed based upon the mixture properties of the exhaust gas as detailed in Section 3.1.2. Generally, the parameters ${\dot{m}}_{11},\omega_{8},h_{5}$ and *h*_8_ are not known and require solving the mass, energy, and exergy balances. One option to find these variables is to solve the internal transport mechanism of the air saturator to yield the outlet temperature and humidity. Additionally, the air saturator has two parts namely upper and lower. Therefore, a control volume‐based heat transfer model is presented by the authors which are solved numerically to yield the outlet parameters. These equations are^[^ ^40^ ^]^:

(42) $$\frac{dT_{wa-dry}}{dx}=\frac{h_{c,wa-dry}\times b}{{\dot{m}}_{wa-dry}c_{p-wa-dry}}\left(T_{wa-dry}-T_{wa-wet}\right)$$

(43) $$\begin{array}{ccc} \frac{dT_{wa-wet}}{dx} & = & \frac{b}{{\dot{m}}_{wa-wet}\times c_{p-wa-wet}}\left[h_{c,wa-wet}\left(T_{wa-dry}-T_{wa-wet}\right)\right.\\ & & \left.+\;h_{m-wa-wet}i_{v}\left(\omega_{saturated}-\omega_{wa-wet}\right)\right] \end{array}$$

(44) $$\frac{d\omega_{wa-wet}}{dx}=\frac{h_{m-wa-wet}\times b}{{\dot{m}}_{wa-wet}}\left(\omega_{s-wet}-\omega_{wa-wet}\right)$$

The upper part of the air saturator is a heat exchanger going through a sensible heat transfer; therefore, the effectiveness‐NTU method is applied which can be consulted in the author's previous work.^[^ ^40^ ^]^

#### 3.1.7 Bottoming Turbine

The mass balance:

(45) $${\dot{m}}_{8}={\dot{m}}_{9}$$

The energy balance:

(46) $${\dot{W}}_{BT}=\dot{E}n_{8}-\dot{E}n_{9}$$

Work of bottoming turbine is multiplied with η_*generator*_η_*mechanical*_. Equation (46) is solved carefully when there could be a possible water condensation in the bottoming turbine, and for such a case, the expansion enthalpy and mass flow rate of water in liquid (because of condensation) form is added with the expansion enthalpy and mass flow rate of humid air in the exit turbine. In other words, the work of the bottoming turbine includes a correction by separately evaluating the work from the fluid flow in the gaseous state and the liquid state.

The exergy balance:

(47) $$\dot{E}x_{xd,BT}=\dot{E}x_{8}-\dot{E}x_{9}-{\dot{W}}_{BT}$$

#### 3.1.8 Evaluation of Thermodynamic Properties

The specific exergy of working fluids (air, exhaust gases, humid air, and water) are calculated using the following equations^[^ ^62^, ^69^, ^73^, ^74^, ^75^ ^]^:

(48) $$ex=\left(h-h_{0}\right)-T_{0}\;\left(s-s_{0}\right)$$

(49) $$\begin{array}{ccc} ex_{ha} & = & \left(c_{p,da}+\omega c_{p,v}\right)T_{0}\left[\frac{T}{T_{0}}-1-\ln\frac{T}{T_{0}}\right]+\left(1+\bar{\omega }\right)R_{da}T_{0}\ln\frac{P}{P_{0}}\\ & & +\;R_{da}T_{0}\left[\left(1+\bar{\omega }\right)\ln\left(\frac{1+{\bar{\omega }}_{0}}{1+\bar{\omega }}\right)+\bar{\omega }\ln\left(\frac{\bar{\omega }}{{\bar{\omega }}_{0}}\right)\right] \end{array}$$

where *T* and *P* are the temperature and pressure of the thermal machine where exergy flow is evaluated. Shukuya and Hammache^[^ ^75^ ^]^ have mentioned that the following exergy flow equation *ex* = (*h* − *h*_0_) − *T*_0_ (*s* − *s*_0_) can be used for water in a compressed state. *R_da_* is the specific ideal gas constant of dry air. $\bar{\omega }$ is the mole fraction ratio which represents the number of moles of water corresponding to 1 mole of dry air in the mixture. One of its approximations is given by $\bar{\omega }≅\left(1.608\right)\omega$.^[^ ^62^, ^69^ ^]^ ${\bar{\omega }}_{0}$ is molar humidity ratio of reference air and it is given by^[^ ^62^, ^69^ ^]^:

(50) $${\bar{\omega }}_{0}≅\left(1.608\right)\omega_{0}$$

while ω_0_ is the absolute humidity at dead state temperature and pressure conditions calculated using^[^ ^76^ ^]^:

(51) $$\omega_{0}=\frac{0.622}{\left(P_{0}/\phi_{0}P_{sat(T_{0})}\right)-1}$$

### 3.2 Performance Indicators

The energy and exergy efficiencies of the overall system are given in the following equations, respectively:

(52) $$\eta_{\mathrm{energy}\mathrm{efficiency}}=\frac{\left[{\dot{W}}_{TT}-{\dot{W}}_{TC}\right]+\left[{\dot{W}}_{BT}-{\dot{W}}_{BC}\right]-{\dot{W}}_{P}}{\dot{E}n_{fuel}}$$

(53) $$\eta_{\mathrm{exergy}\mathrm{efficiency}}=\frac{\left[{\dot{W}}_{TT}-{\dot{W}}_{TC}\right]+\left[{\dot{W}}_{BT}-{\dot{W}}_{BC}\right]-{\dot{W}}_{P}}{\dot{E}x_{fuel}}$$

The component‐wise entropy generation rate $\left({\dot{S}}_{i}\right)$ and the relative exergy destruction $\left(\dot{E}x_{xd,ratio,i}\right)$, respectively are given by:

(54) $${\dot{S}}_{i}=\frac{\dot{E}x_{xd,i}}{T_{0}}$$

(55) $$\begin{array}{c} \dot{E}x_{xd,ratio,i}=\frac{\dot{E}x_{xd,i}}{\dot{E}x_{xd,TC}+\dot{E}x_{xd,CC}+\dot{E}x_{xd,TT}+\dot{E}x_{xd,BC}+\dot{E}x_{xd,P}+\dot{E}x_{xd,AS}+\dot{E}x_{xd,BT}} \end{array}$$

### 3.3 Solution Methodology

**Figure 2:**
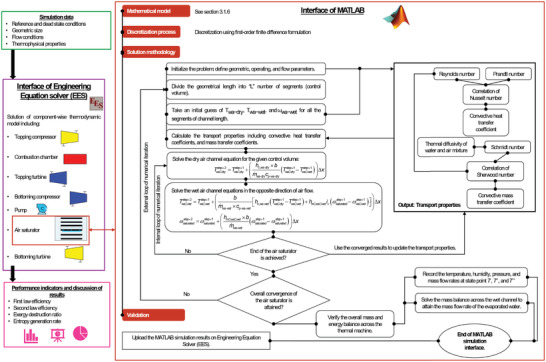
Integrated methodology diagram for the solution of energy and exergy balances on the global component level.

The integrated methodology diagram consists of component level energy balance (here called “global energy balance”) with the heat transfer model of the air saturator. A stepwise procedure is also detailed in **Figure** **2**.

It consists of different steps starting from the simulation data (**Table** **2**) to solving the thermodynamic balances. However, the thermodynamic balances cannot be successfully completed, because, properties at the outlet of the air saturator are not known.

**Table 2:** Simulation Parameters.

Parameter	Value	Reference
${\dot{m}}_{1}$	94.8 kg/s	[38]
$P_{2}/P_{1}$	11.8	[43]
${\dot{m}}_{fuel}$	2.22 kg/s	[38]
*T*_3_	1545 K	‐
*LHV*	47.1 MJ/kg	[89]
Pressure drop in the combustion chamber	5%	[37]
η_*s*,*TC*_, η_*s*,*BC*_, η_*s*,*TT*_, η_*s*,*BT*_	0.85	[45]
η_*s*,*P*_	0.90	[45]
*T*_10_	298 K	[37]
Pressure drop in each channel of air saturator	2.5%	‐
$P_{7}/P_{6}$	2.45	‐
${\dot{m}}_{6}/{\dot{m}}_{1}$	0.775	‐

Two methods are available to evaluate the outlet properties of the air saturator (temperature, humidity, pressure, and water injection rate). The first one is also to apply an overall mass and energy balance at the boundary of the air saturator like the one published by Saghafifar et al.^[^ ^44^ ^]^ However, this method has its own limitations because several extra parameters are needed like air saturator degree of humidification/saturation, dewpoint, or wet‐bulb effectiveness of air saturator, etc. These parameters should be a part of the mathematical model itself because they have a dependency on the geometric, operational, and flow conditions of the air saturator. Consequently, a mathematical model is required which consists of the air saturator physics and its corresponding heat transfer characteristics.

For this purpose, the authors have developed a mathematical model considering a set of appropriate assumptions. A control volume is selected which consisted of a dry air channel, and a wet air channel (containing humid air and water film). Energy balance equations are applied on each channel of the air saturator (humidification section only) in which the heat balance is written in terms of the appropriate mode of heat transfer. For the case of dry air, only the sensible heat transfer to the adjacent wet channel is considered. The rate of heat transfer is determined by the appropriate convective heat transfer coefficient (Figure 2; Equation 43). Since the absolute humidity content of the dry air remains the same, therefore, it follows an equality mass conservation principle at the inlet and outlet. In the case of wet air, mass and energy balance are applied. The mass balance is based upon Fick's law and considers a convective mass transfer coefficient of evaporating water into the adjacent wet air (Equation 44). The energy balance is written considering the sensible heat change, and the latent energy (water evaporation) (Equation 43). These equations are discretized using first‐order finite‐difference formulation and written in terms of the desired variable in the next space step.

The solution of this mathematical model is “somewhat” complicated because it offers a coupled boundary condition problem. The dry air is regenerated to the wet channel at the end of the air saturator. Therefore, the solution of the first control volume is not possible because the wet air quality (temperature, and humidity) are unknown. Additionally, the problem is also coupled in terms of its own variables. For example, the temperature and humidity conditions of the entire channel are required to estimate thermal transport characteristics. Moreover, it is also not known that a particular number of control volumes would provide convergence to the entire air saturator assembly.

To address these complications in the numerical formulation, the authors have developed an intelligent manner to integrate different iteration procedures. First, the channel length is divided into a specific number of control volumes. The temperature and humidity properties of all the working fluids are assumed to start the simulation. The thermal transport characteristics are calculated based upon the assumed temperature and humidity properties. In this step, the dry air equation is solved for the first control volume until reaching the end of the air saturator. Thus, in this way, there are updated dry air temperatures including the boundary conditions. These updated dry air temperatures are used to solve the wet air temperature and humidity equations until reaching the exit of the air saturator again. In this way, the first iteration step is completed. Based upon the updated energy balance, the thermal transport properties are re‐calculated, and the process is continued until an error of 0.001% is noted among the successive iterations. This iteration step is called the “internal loop of numerical iteration” as in Figure 2. However, it is not sufficient to establish the convergence of the entire grid. For this, the number of control volumes per air saturator is increased, and the “internal loop of numerical iterations” is carried out again. Finally, a grid (length of a control volume: 0.05 m) is selected once the successive grids attain the same results.

The thermodynamic model is solved on Engineering Equation Solver (EES)^[^ ^77^ ^]^ and the thermophysical properties are extracted from the same software. The details of the thermophysical properties of each of the working fluids in the configuration are as follows:

1. The thermodynamic and transport properties of dry air are implemented assuming that air obeys the ideal gas law. Thus, using the ideal gas relations, it is only necessary to obtain information that how the specific heat varies with temperature in order to determine all the thermodynamic properties. For the temperatures between 100 K and 2000 K, the air property routine uses the ideal gas‐specific heat relation using the work of Lemmon et al.^[^ ^78^ ^]^ For temperatures between 2000 K and 3500 K, the thermodynamic properties are based upon the data from Kennan et al.^[^ ^79^ ^]^ The transport property correlations are based on the tabular data available from the references.^[^ ^80^, ^81^ ^]^ The range of validity of the viscosity correlation is between 80 K and 2000 K. The range of the correlation of thermal conductivity is between 80 K and 1500 K. It is also pertinent to mention that the reference value used for the entropy of air may differ from the property tables in the standard thermodynamic textbooks. However, the interest in thermodynamic correlations is the difference between the entropy, and in this way, the reference value cancels, and its absolute value does not matter.
2. The thermodynamic properties for carbon dioxide (CO_2_) are also based upon ideal gas law which is valid over a temperature range from 200 K to 3500 K based on the property information from Mcbride et al.^[^ ^82^ ^]^ The reference state for specific enthalpy is based on the enthalpy of a formation relative to the elements at 25 °C. The reference state for specific entropy is based on the Third Law of Thermodynamics. Transport property correlations are implemented using the data provided by Vargaftik et al.^[^ ^83^ ^]^ The viscosity and thermal conductivity correlation are applicable for an operating temperature ranging between 150 K to 2000 K, and 200 K to 1400 K, respectively.
3. The thermodynamic properties for nitrogen gas (N_2_), oxygen gas (O_2_), and gaseous water (H_2_O_(g)_) is also based upon ideal gas thermodynamic properties information provided by McBride et al.^[^ ^82^ ^]^ valid over the temperature range from 200 K to 3500 K. The reference state for specific enthalpy and specific entropy is based upon the enthalpy of a formation relative to the elements at 25 °C and Third Law of Thermodynamics, respectively. Transport property calculations are implemented from the data provided by Vargaftik^[^ ^83^ ^]^ in which the correlation of viscosity is applicable for a temperature range between 80 K and 2500 K, and thermal conductivity correlation is applicable between 80 K and 1400 K.
4. The humid air mixture (Air‐H_2_O) consists of the air‐water vapor mixture (psychrometric) properties using thermodynamic data from the built‐in libraries of air and steam property relations from Hyland and Exler.^[^ ^84^ ^]^ Wet‐bulb temperature is estimated using the adiabatic saturation temperature. The reference state point of the specific enthalpy of dry air is 0 °C as consistent with most psychrometric charts. Specific entropy is referenced at 0 K to be consistent with air. The reference state for both enthalpy and entropy of water vapor is based on the traditional steam table reference state. The properties of air‐water vapor mixtures are calculated assuming the ideal gas behavior. Viscosity and thermal conductivity of air‐water vapor mixture are determined using the transport property data for pure air and pure water vapor mixing results as described by Van den Bulck.^[^ ^85^, ^86^ ^]^

The platform of Engineering Equation Solver (EES) is not user‐friendly to carry out such a looped iterative procedure. Therefore, the programming is carried out in an integrated platform of EES and MATLAB.^[^ ^87^ ^]^ The component‐wise energy balance is solved on the EES interface. The inlet conditions of the EES program are used on MATLAB and the results are fed back to the EES code. To maintain the consistency in the thermophysical properties, a dataset of each of the desired properties is extracted which is curve fitted using statistical package STATGRAPHICS.^[^ ^88^ ^]^ It is also pertinent to mention that each correlation fulfills the statistical criteria through the indicators like the coefficient of determination, mean absolute percentage error, and root means square error.

The authors have adapted various verification procedures to justify the validity of the adapted model and the methodology. Unfortunately, there is still no experimentation available on this cycle, therefore, the only way to verify the validity of this model is through a comparison with other studies at a component‐scale (topping cycle and air saturator) and on a general scale. It is done in various steps.

1. In the first step, the gas turbine topping cycle is validated. The authors took the design and operating parameters of the actual SIEMENS GT‐800 gas turbine.^[^ ^90^ ^]^ The analysis data is as follows: compression ratio equal to 21.1, turbine inlet temperature equal to 1500 K, exhaust gas temperature equal to 826 K, air mass flow rate equal to 131.54 kg s^−1^, and exhaust gas mass flow rate equal to 134.2 kg s^−1^. The authors have calculated the fuel mass flow rate, power output, and first law thermal efficiency which came out to be 2.79 kg s^−1^, 53.85 MW, and 40.68%, respectively. The fuel mass flow rate, power output, and first law thermal efficiency of SIEMENS GT‐800 gas turbine^[^ ^90^ ^]^ are 2.67 kg s^−1^, 50.5 MW, and 38.30%, respectively. Based on this, the error ranges between 4.49% to 6.21% in all the indicators which validate the model of the topping cycle. These details can be seen further in the author's previous work.^[^ ^40^ ^]^ A similar validation study is also conducted by Saghafifar et al.^[^ ^91^ ^]^
2. In the second step, the authors have benchmarked the model of the lower part of the air saturator, i.e., the M‐cycle cooling device. For this purpose, standard atmospheric conditions were considered for the operation of the air saturator, and the experimentally conducted parameters of Riangvilaikul et al.^[^ ^92^, ^93^ ^]^ are modeled. It contains the following data for analysis purposes: channel length equal to 1200 mm, channel width equal to 80 mm, channel height equal to 5 mm, working to intake air ratio equal to 0.33 kg kg^−1^, and flow rate of feed water equal to 60 g h^−1^. In the next step, two different kinds of experiments are conducted. In the first one, the inlet air temperature is varied from 25 °C to 45 °C on different levels of inlet absolute humidity (6.9, 11.2, 20.0, and 26.4 g kg^−1^) and the outlet air temperature is noted. In the second kind of experiment, the air velocity in the channel is varied from almost 1.5 m ^−1^s to 6 m ^−1^s on two different inlet conditions (34 °C and 19.0 g kg^−1^, and 34 °C and 11.2 g kg^−1^) whereas the outlet air temperature is noted. The simulation conditions are carefully used in the air saturator model and finally, the simulation results are plotted against the experimental results of Riangvilaikul et al.^[^ ^92^, ^93^ ^]^ A graphical description of this comparison can be seen in the author's previous work.^[^ ^94^ ^]^ In this way, strong conformity between this experimental study and the simulation study also verified the modeling procedure of the air saturator.
3. In the third step, the authors compared the thermal efficiency of the overall thermodynamic cycle with the parameters used by Saghafifar et al.^[^ ^44^ ^]^ It included the usage of extraction ratio (regeneration ratio) equal to 0.33, air saturator degree of humidification equal to 0.55, turbine inlet temperature equal to 1400 K, topping cycle pressure ratio equal to 14, bottoming cycle pressure ratio equal to 4, and mass flow rate ratio equal to 1.0. The efficiency of Saghafifar et al.^[^ ^44^ ^]^ is 33.5% and for the current study is 32.6% which is within the 10% conformity rank of both studies.

## 4 Results and Discussion

### 4.1 Sensitivity of Air Saturator Split (Extraction) Ratio

The split ratio inside the air saturator has key importance. It determines the role of the upper and lower part of the air saturator. Therefore, finding its optimal value by energetic and exergetic indicators is very important before reporting the results.

**Figure 3:**
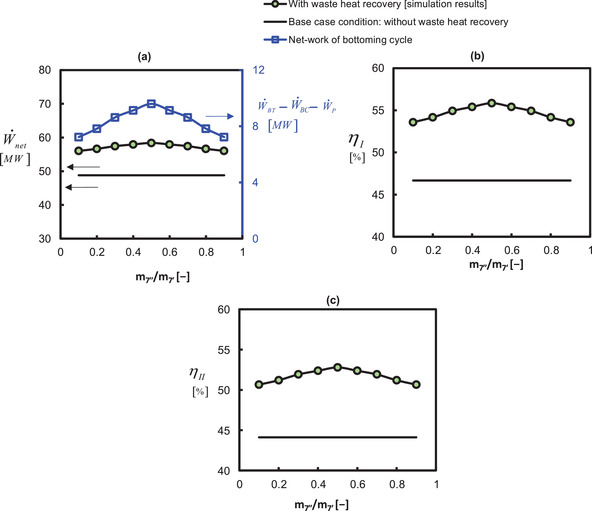
Influence of air saturator split ratio on a) work output, b) first law efficiency, and c) second law efficiency. Note: The base case condition consists of a simple gas turbine without its integration to any waste heat recovery unit and it is labeled as “without waste heat recovery”. η_*I*_ refers to the first law energy efficiency(η_energyefficiency_), and η_*II*_refers to the second law exergy efficiency (η_exergyefficiency_).

**Figure** **3**a–d presents the sensitivity of split (extraction) ratio on bottoming cycle net‐work output $\left({\dot{W}}_{BT}-{\dot{W}}_{BC}-{\dot{W}}_{P}\right)$, net‐work output $\left({\dot{W}}_{TT}-{\dot{W}}_{TC}+{\dot{W}}_{BT}-{\dot{W}}_{BC}-{\dot{W}}_{P}\right)$, energetic $\left(\eta_{\mathrm{energy}\mathrm{efficiency}}=\frac{\left[{\dot{W}}_{TT}-{\dot{W}}_{TC}\right]+\left[{\dot{W}}_{BT}-{\dot{W}}_{BC}\right]-{\dot{W}}_{P}}{\dot{E}n_{fuel}}\right)$ and exergetic efficiencies$\left(\eta_{\mathrm{exergy}\mathrm{efficiency}}=\frac{\left[{\dot{W}}_{TT}-{\dot{W}}_{TC}\right]+\left[{\dot{W}}_{BT}-{\dot{W}}_{BC}\right]-{\dot{W}}_{P}}{\dot{E}x_{fuel}}\right)$. The maximum ${\dot{W}}_{net}$, ${\dot{W}}_{BT}-{\dot{W}}_{BC}-{\dot{W}}_{P}$, η_*I*_, and η_*II*_ are 58.39 MW, 9.593 MW, 55.85%, and 52.79%, respectively is noted for a split (extraction) ratio of 50%.

A lower value of the split ratio $\left({\dot{m}}_{7^{\prime \prime }}/{\dot{m}}_{7^{\prime }}\right)$ corresponds to a lower mass fraction of air in the wet channel $\left({\dot{m}}_{7^{\prime \prime }}\right)$ and a higher mass fraction of air in the upper part of the air saturator signifying a lower water evaporation/injection in the wet channel. In this way, the humidity content at the inlet of bottoming turbine would be lower and the bottoming cycle resembles a dry air waste heat recovery cycle which has a lower potential of generating power than the humid air cycle. Owing to this, the performance indicators are lower for a lower split ratio. Nevertheless, the performance indicators are increasing with the increase in the split ratio. Because the role of the wet channel is increasing which indicates a higher water injection due to the more availability of air in the wet channel. It also corresponds to a lower mass flow rate to recover the heat from the topping cycle. In other words, at a higher split ratio, the role of the upper part of the air saturator is also decreasing. It signifies that the capacity of the bottoming cycle to absorb the waste heat from the topping cycle is decreasing. In conclusion, a split ratio of 0.5 is optimal which addresses an equivalent role of the upper and lower parts of the air saturator supplying the suitable humidity and temperature to the bottoming turbine.

### 4.2 Sensitivity of Mass Flow Rate Ratio and Pressure Ratio

**Figure 4:**
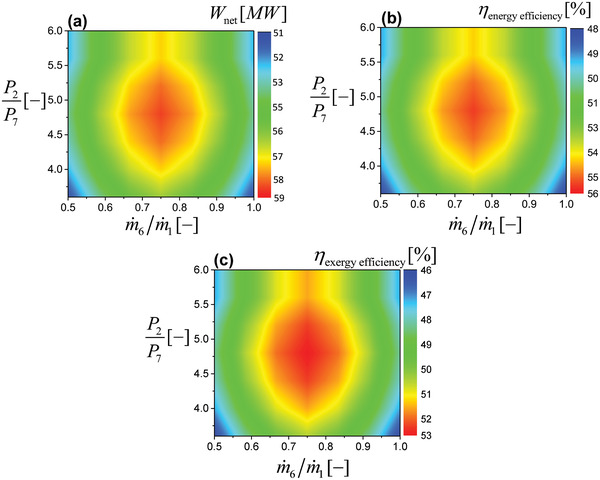
Influence of mass flow rate ratio and pressure ratio on a) work output, b) first law efficiency, and c) second law efficiency.

Other important tuning parameters are the mass flow rate ratio $\left({\dot{m}}_{6}/{\dot{m}}_{1}\right)$ and pressure ratio $\left(P_{2}/P_{7}\right)$ between the topping and the bottoming cycles. The sensitivity of these parameters with respect to the performance indicators is presented in **Figure** **4**. The process optimality of the waste heat recovery unit exists in the most suitable combination of mass flow rate ratio $\left({\dot{m}}_{6}/{\dot{m}}_{1}\right)$ and pressure ratio $\left(P_{2}/P_{7}\right)$. In other words, it is desired to evaluate the optimality of the operation which provides the information about the best values of the performance indicators.

A lower mass flow rate ratio $\left({\dot{m}}_{6}/{\dot{m}}_{1}\right)$ indicates a higher mass content in the topping cycle $\left({\dot{m}}_{1}\right)$ as compared to the bottoming cycle $\left({\dot{m}}_{6}\right)$. The performance indicators are lower because of the unavailability of sufficient air in the bottoming cycle to recover the waste heat. As expected, the performance indicators are increasing with the increase of air in the bottoming cycle, because, more content of air is available to accept waste heat, and consequently the water vapor elevates the work output of the bottoming turbine. The performance parameters are maximum for a mass flow rate ratio between the range of 0.7 to 0.8. Nevertheless, the performance indicators are decreasing sharply after this range. Because, very high mass content is available in the bottoming cycle; surpassing the topping cycle, becoming less favorable to accept the measured quantity of waste heat.

In the same perspective, a pressure ratio equal to the unit corresponds to the equality of topping and bottoming pressure signifying a lower potential to accept the waste heat. As the pressure ratio is increasing, the bottoming pressure is becoming lower as compared to the topping cycle which is allowing extracting the work of the bottoming turbine until reaching the optimum range of pressure ratio between 2.5 to 2.7 and decreasing afterward. The decrement of pressure ratio after this range is supported by the argument that after this range, the bottoming pressure decreases significantly which corresponds to a lower moisture absorption capability of air.

The design of the result section is carried out into two sections. In the first part, the optimal air saturator split (extraction) ratio is calculated and in the second part, the optimal mass flow rate ratio and pressure ratio are attained through the usage of a constant (optimal) air saturator split (extraction) ratio. However, it would be interesting to understand that how the operational optimality of mass flow rate ratio and pressure ratio would be influenced by changing the optimal air saturator split (extraction) ratio. The answer to this would exist in a hypothetical four‐dimensional composite curve having air saturator split (extraction) ratio in the z‐axis, mass flow rate ratio in the x‐axis, pressure ratio in the y‐axis, and the performance indicator on the fourth axis. Various sample tests are carried out to understand the comportment of this composite curve. It is deduced that the optimal contour of the composite curve exists at the air saturator split (extraction) ratio of 0.5 and all other contours for an air saturator split (extraction) ratio less than 0.5 or greater than 0.5 remain below the optimal level of air saturator split (extraction) ratio. In this sense, it can be inferred that the findings of Figure 4 correspond to the optimal contour level of the air saturator split (extraction) ratio for the mentioned performance indicators. In conclusion from this section, a mass flow rate ratio of 0.7 to 0.8 (precisely to 0.75), a pressure ratio of 3.6 to 6 (precisely to 4.8), and an air saturator split (extraction) ratio of 0.5 are optimal for the performance indicators.

### 4.3 Overall Plant Performance

**Figure 5:**
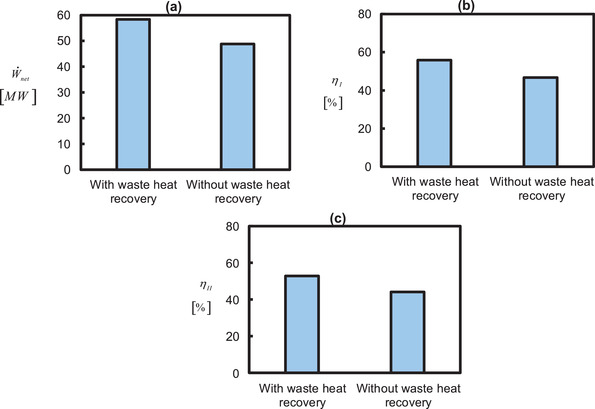
a) Net‐work output rate, b) energy efficiency, and c) exergy efficiency for optimal parameters: ${\dot{m}}_{7^{\prime \prime }}/{\dot{m}}_{7^{\prime }}=0.5;\;P_{2}/P_{7}=4.8;\;{\dot{m}}_{6}/{\dot{m}}_{1}=0.75$. Note: The base case condition consists of a simple gas turbine without its integration to any waste heat recovery unit and it is labeled as “without waste heat recovery”. η_*I*_ refers to the first law energy efficiency (η_energyefficiency_), and η_*II*_refers to the second law exergy efficiency (η_exergyefficiency_).

The energetic and exergetic performance indicators of the overall system are presented in **Figure** **5**. The maximum rate of work generated by the system for the optimized ${\dot{m}}_{6}/{\dot{m}}_{1},\;P_{2}/P_{7}$ and ${\dot{m}}_{7^{\prime \prime }}/{\dot{m}}_{7^{\prime }}$ is 58.39 MW which represents a ≈20% advantage in power production to include a bottoming cycle in the gas turbine topping cycle. The energetic and exergetic efficiencies are 55.85% and 52.79%, respectively, which are also ≈20% higher than the case of the gas turbine topping cycle alone. The energy efficiency is higher than the exergy efficiency because the chemical exergy factor φ is greater than one (1.058) which gives $\dot{E}n_{fuel}<\dot{E}x_{fuel}$, thus, the exergy efficiency $\eta_{\mathrm{exergy}\mathrm{efficiency}}=\frac{\left[{\dot{W}}_{TT}-{\dot{W}}_{TC}\right]+\left[{\dot{W}}_{BT}-{\dot{W}}_{BC}\right]-{\dot{W}}_{P}}{\dot{E}x_{fuel}}$ becomes smaller than the energy efficiency $\eta_{\mathrm{energy}\mathrm{efficiency}}=\frac{\left[{\dot{W}}_{TT}-{\dot{W}}_{TC}\right]+\left[{\dot{W}}_{BT}-{\dot{W}}_{BC}\right]-{\dot{W}}_{P}}{\dot{E}n_{fuel}}$ knowing that a higher denominator is divided in the exergy efficiency formula for the same numerator in both cases.

### 4.4 Entropy Generation Rate, Relative Exergy Destruction, and Exergy Distribution

**Figure 6:**
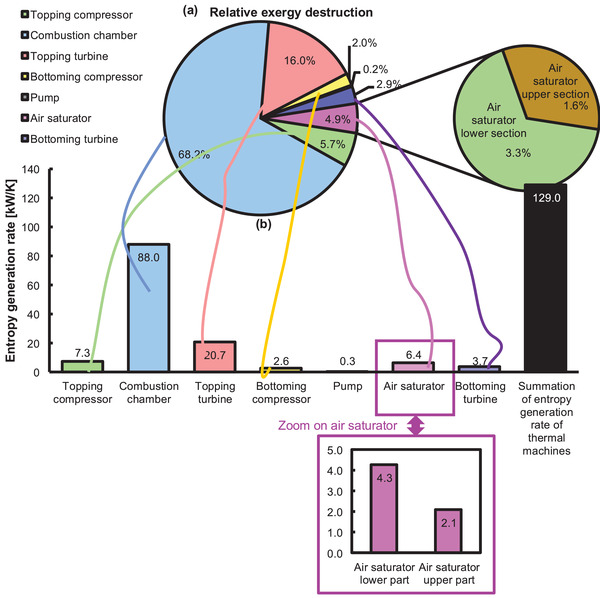
a) Relative exergy destruction, and b) entropy generation rate of the system (excluding the outwards exergy flow from steam 5 and 9).

**Figure** **6**a,b presents the entropy generation rate and the exergy destruction ratio of all the components of the power generation unit. The highest relative exergy destruction and entropy generation rate are found for the combustion chamber, topping turbine, topping compressor, air saturator, bottoming turbine, bottoming compressor, and pump. It is quite expected that the combustion chamber has the highest relative exergy destruction and entropy generation rate owing to the combustion process. The topping turbine has the second‐highest relative exergetic destruction because the state point at the exit of the turbine is designed to be utilized to run the bottoming cycle; and even though it is reutilized in the bottoming cycle, nevertheless, a large amount of exergy leaves the system in the form of stream 5. It is a common concept that the pumping work is neglectable in a combined cycle power plant; in this case, the pump contributes to an entropy generation rate of 0.3 kW K^−1^, because it is used for injection purposes in a high‐pressure air saturator.

The entropy generation rate and exergy destruction of the bottoming turbine are also significant. It can be minimized by reutilizing it for any poly‐generation processes. The exergy destruction ratio in the air saturator is 6.1%, which is high as compared to the exergy destruction ratio of the air saturator calculated by Caliskan et al.^[^ ^43^ ^]^ (around ≈3%). Because, in the current work, the air saturator has two sections: the upper and the lower part, and the exergy destruction ratio of 6.1% corresponds to the irreversibilities in both sections. Additionally, current work considered the inside transport mechanisms of the air saturator. The usefulness of this work including the heat and mass transfer characteristics of the air saturator can yield a more realistic exergy destruction ratio of the air saturator.

**Figure 7:**
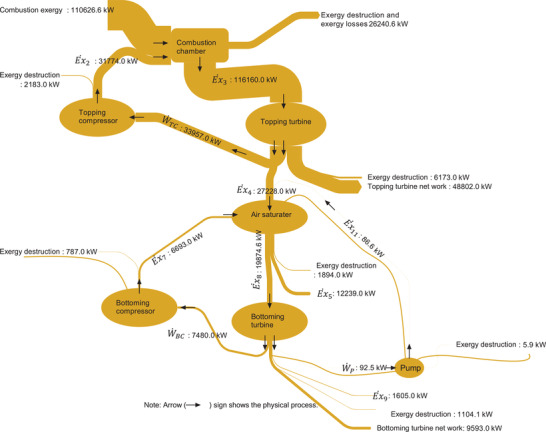
Grassmann diagram showing the exergy distribution of the combined cycle power plant.

It is also pertinent to mention that the relative exergy destruction and the entropy generation rate of the air saturator seems to be much less considering the situation that fluid at relatively high thermodynamical $\left({\dot{m}}_{5}=96.98\frac{kg}{s},T_{5}=646.5K,P_{5}=106.7kPa\right)$ characteristics are leaving the air saturator. It is because the entropy generation rate of any thermodynamic machine is calculated based upon its exergy destruction which comes up from the exergy balance, i.e., subtracting the flow losses as shown in the exergy balance of the air saturator $\dot{E}x_{xd,AS}=\dot{E}x_{7}+\dot{E}x_{4}+\dot{E}x_{11}-\dot{E}x_{5}-\dot{E}x_{8}$. With this perspective, it can be said that the flow exergy from state 5 $\left(\dot{E}x_{5}\right)$ is significant $\dot{E}x_{5}=12239.0kW$ (see **Figure** **7**), and indirectly, the balance has accumulated it.

Another point of thought is to review in‐depth relative exergy destruction rate and the entropy generation rate within the air saturator. It can be seen in Figure 6 that the relative exergy destruction contribution towards the total cycle for the lower section and upper section are 3.3% and 1.6%, respectively. It signifies that the lower section of the air saturator takes ∼67% (1270.8 kW) of the relative exergy destruction of the complete air saturator (1894 kW) whereas the upper section of the air saturator contributes to only ≈33% (623.12 kW). Similar percentages are also noted for the entropy generation rate between the upper and the lower section of the air saturator. Thus, it can be said that the entropy generation rate and the relative exergy destruction of the lower section of the air saturator are higher than the upper section of the air saturator. It is because the upper section is simply a heat exchanger to recover the waste heat from the gas turbine topping cycle. It is different from the lower section where additional fluids (injection of water) and operations (dry air regeneration and evaporation processes) are contributing towards the entropy generation rate and relative exergy destruction. Therefore, within the air saturator, the lower section is more critical towards the optimization studies for the minimization of exergy destruction.

Figure 7 shows an extremely vital finding of the article which displays the exergy distribution of the entire combined cycle power plant in the form of a Grassmann diagram. The exergy flow from the topping compressor is 31 774 kW and the exergy destruction is 2183 kW. The exergy of the pressurized ambient air is combusted with an inflow combustion exergy of 110 626.6 kW. In the combustion chamber, there is exergy destruction and exergy loss of 26 240.6 kW which is the highest among all the thermal machines. The turbine generates a total work of 82 759 kW in which 33 957 kW are consumed by the topping compressor leaving only 48 802 kW as the net power of the topping cycle. The topping turbine has exergy destruction of 6173 kW.

In the bottoming cycle, the exergy of 27 228 kW, 86.8 kW, and 6693 kW enters the air saturator corresponding to the flow exergy of the topping cycle, bottoming pump, and the bottoming compressor. In this way, the air saturator has an outlet useful flow exergy of 19 874.6 kW which is supplied to the bottoming turbine; however, its exergy destruction is around 1894 kW. Similarly, a huge quantity (12 239.0 kW) of exergy flows out of the air saturator in the form of exhaust gases after dumping its heat into the air saturator. In the bottoming turbine, the exergy of 7480 kW and 92.5 kW is supplied to the bottoming compressor and the pump, with exergy destruction of 1605.1 kW, leaving only 9593.0 kW as the useful work of the bottoming turbine.

Another important and interesting perspective is related to the pumping algorithms and patterns for the efficient management of the humid air turbine cycle. The humid air turbine cycle has a continuous operation and there can be perturbations and uncertainties in the air‐water mixture properties within the air saturator. In such a case, it is necessary to have excess injected water into the casing of the air saturator which can be readily available in case of any perturbation in the water evaporation process. Normally, this excess water is neglected for the energy balance because it enters and leaves the air saturator at the same temperature. However, it can be crucial for the pumping power of the pump. Paepe et al.^[^ ^32^ ^]^ also outlined some difficulties related to the water injection. The configuration of Paepe et al.^[^ ^32^ ^]^ has injected the water after the compressor and mentioned that the injection of water can cause combustion instability which can lead to increased environmental emissions (unburned hydrocarbon, and CO). In fact, Hermann et al.^[^ ^95^ ^]^ has recommended that the maximum limit for the water injection before the combustion chamber is 33 weight percent of water in the gases because the CO levels increase after this limit owing to the instabilities in combustion. The excess water injection also corresponds to high water treatment costs because the condensed water, along with other environmental contaminants, can have an acidic nature which can damage the metallurgical limitations of the gas turbine. There can be another debate to this discussion that what would be the optimal excess water injection flow rates in the air saturator. In future work, dynamic and transient simulations can be performed with different excess water percentages along with the operational perturbations of the evaporation process to yield a robust optimization scenario of the excess water injection.

The water is only injected in the wet section which only represents 50% (≈36.7 kg s^−1^) of the total mass flow rate in the bottoming section. The pressure of the humid air before the bottoming turbine is high (230.1 kPa) which indicates that the air at this state has a high capability to retain the moisture in its dry air fraction. The problem occurs when this humid air is expanded in the bottoming turbine where its pressure is dropped. This loss of pressure also decreases the capability of the humid air to retain the same amount of water vapor content in the air. Consequently, it is expected to have a high condensation of the water in the bottoming turbine. One option to avoid this is to heat the air before it can enter the bottoming turbine, which is in fact, a subpart of this waste heat recovery unit where the air is heated in the upper part of the air saturator. This, somehow, mitigates the possible failure induced by the condensation at the bottoming turbine. Another perspective of this discussion is related to the design of the turbomachinery. Several conventional turbine systems can run on a certain level of water humidity in the working fluid which helps to mitigate its environmental impacts (especially NOx emissions and particulate matter), but this problem is more complicated for Maisotsenko humid air turbine cycles. The usage of this advanced configuration of air saturator is beneficial from the aspects of enhanced air humidification before the inlet of the bottoming turbine, but it is also possible that the usage of enhanced air humidification breaks the physical barriers and limitations of turbomachinery's metallurgy of conventional gas turbines. Thus, in this case, it is necessary to carry out further research in this area on the engineered design of the turbines with such a high rate of water content in their operational working fluid. It can also be correlated with the pressure distribution of the working fluid within the stages of the bottoming turbine. In the stages of the beginning of the compressor, the condensation would not be an issue because the pressure still is high enough to avoid the condensation. However, this problem can get severe as the pressure keeps on dropping across the working direction of the turbine. Thus, in this case, it would only be enough to use advanced metallurgical characteristics of only the last stages of the gas turbine.

## 5 Conclusion

The humid air bottoming cycle can be one of the candidates for the waste heat recovery in a gas turbine topping cycle. In this work, energy, and exergy analyses, coupled with a heat transfer model of air saturator, of such a waste heat recovery unit are needed to be analyzed for the cleaner technical process assessments. Followings are the key findings of the work:

- The optimal mass flow rate ratio $\left({\dot{m}}_{6}/{\dot{m}}_{1}\right)$ between the bottoming and the topping cycle ranges between 0.7 to 0.8 considering the energetic and exergetic performance indicators.
- The optimal pressure ratio $\left(P_{2}/P_{7}\right)$ between the topping and bottoming cycle ranges between 4.5 to 5.0 considering the energetic and exergetic performance indicators.
- The optimal air saturator split ratio between the upper part and the lower part of the air saturator is 0.5 considering the energetic and exergetic performance indicators.
- The maximum work generation is 58.39 MW, energy efficiency is 55.85%, and exergy efficiency is 52.79%. The exergy analysis shows that this system has higher energy efficiency as compared to exergy efficiency. So, it is important to calculate the exergetic dimensions of this kind of thermal system to reach more reliable results.
- Combustion chamber, topping turbine, topping compressor, and air saturator are the key components having the highest exergy destruction ratio as 68.2%, 16.0%, 5.7%, and 4.9%, respectively; while the corresponding entropy generation rates are 88.0, 20.7, 7.3, and 6.4 kW K^−1^, respectively. So, these components are the major elements to be optimized first for a better exergetic ecological performance.

It is concluded that the humid air waste heat recovery alternatives (especially the Maisotsenko cycle based) have energetically and exergetically competitive options as compared to the air bottoming cycles, conventional humid air cycles, and steam‐injected cycles. Theoretically, these modified air saturators are quite attractive for their energetic and exergetic performance and their easiness to integrate with conventional gas turbine topping cycle; however, its practical implementation can be quite challenging especially considering the air sealing mechanisms of the air saturator, because, it is working on the pressure maintained by the bottoming compressor. Additionally, the turbomachinery selection, working on the humid air cycle, can also be challenging. Because condensation can occur at the turbine exit which can be harmful to the metallurgy. Therefore, it is possible that turbomachinery selection and design procedures can be adapted specially for the bottoming turbine. Additionally, the thermoecological footprints of such a waste heat recovery unit need to be analyzed. Nevertheless, these waste heat recovery systems can be made more sustainable through their integration with solar thermal systems. Because, these waste heat recovery units can be operated at relatively lower temperatures; for example, ∼90 °C can be supplied by a concentrating solar collector. Although, there could be complications because of the intermittent nature of solar energy; yet, the usage of efficient thermal storage systems, like phase‐changing material, and/or proposing solar/combustion chamber hybrid schemes can be key. This combined cycle power plant is yet in its initial stages of development and these challenges can be addressed in our future work.

In conclusion, the comprehensive investigation of Maisotsenko's cycle‐based waste heat recovery system in this study not only advances the understanding of gas turbine efficiency and environmental sustainability but also serves as an exemplary didactic resource for the education of budding thermodynamic researchers. By presenting fundamental equations and conducting in‐depth analyses of system performance, this research underscores the significance of innovative approaches in both enhancing technology and nurturing the next generation of thermodynamics experts. The seamless integration of theoretical insights with practical applications paves the way for educational innovation, ensuring that students and researchers alike can contribute to the ongoing development of energy‐efficient solutions and sustainable practices in the realm of thermodynamics.

## Conflict of Interest

The authors declare no conflict of interest.

## Data Availability

The data that support the findings of this study are available from the corresponding author upon reasonable request.

## Appendix A State properties

**Table:**

State	Mass flow rate, kg/s	Temperature, K	Pressure, kPa	Energy rate, kW	Exergy rate, kW
1	94.8	298.2	101.3	28306	0
2	94.8	650.3	1196	62263	31774
Fuel	2.22	‐	‐	104562	110626.59
3	96.98	1546	1136	171140	116160.0
4	96.98	873.8	109.4	88381	272,28.0
5	96.98	646.1	106.7	63658	12239.0
6	73.47	298.2	101.3	21937	0
7	73.47	400.2	248.2	29417	6693
8	211	530	230.1	86024	19874.6
9	211	318.2	109.4	68858.5	1605.0
10	137.5	298.2	9.81^a)^	14410	0
11	137.5	298.2	744.7^b)^	14504	86.6

^a)^Static water pressure of water considering the column height;

^b)^A high water injection pressure is considered for the easiness of operation.

## References

[1] U. Nations , Transforming our world: the 2030 Agenda for Sustainable Development, United Nations Sustainable knowledge platform. 2015.

[2] T. K. Ibrahim , M. M. Rahman , O. M. Ali , F. Basrawi , R. Mamat , M*ATEC Web Conf* 2016, 38, 01002.

[3] E. N. George , M. B. Bailey , W. Domigan , Energy 2010, 35, 1216.

[4] F. Galuppo , T. Reiche , V. Lemort , P. Dufour , M. Nadri , Energy 2021, 216, 119074.

[5] E. Guelpa , V. Verda , Energy 2020, 206, 118024.

[6] Z. Xu , Y. Lu , B. Wang , L. Zhao , Y. Xiao , Energy 2021, 219, 119660.

[7] T. K. Ibrahim , M. K. Mohammed , O. I. Awad , R. Mamat , M. K. Abdolbaqi , MATEC Web Conf 2018, 225, 01020.

[8] F. Basrawi , A. I. M. Al‐Anati , T. K. Ibrahim , M. H. Yusof , A. A. Razak , S. A. Sulaiman , T. Yamada , MATEC Web Conf 2018, 225, 04012.

[9] D. Pashchenko , R. Mustafin , I. Karpilov , Energy 2022, 258, 124913.

[10] M. Montero Carrero , W. De Paepe , S. Bram , A. Parente , F. Contino , Appl. Energy 2017, 204, 1163.

[11] M. Jonsson , J. Yan , Energy 2005, 30, 1013.

[12] W. De Paepe , M. Montero Carrero , S. Bram , A. Parente , F. Contino , J Eng Gas Turbines Power 2018.

[13] M. Cârdu , M. Baica , Energy Convers Manag 2002.

[14] J. J. Lee , M. S. Jeon , T. S. Kim , Appl. Energy 2010, 1307.

[15] S. Motahar , A. A. Alemrajabi , Int. J. Hydrogen Energy 2009, 2396.

[16] H. Zhao , P. Yue , L. Cao , Int J Photoenergy 2009, 672627.

[17] A. D. Rao , US patent no. US4829763A 1989.

[18] M. Nakhamkin , E. C. Swensen , J. M. Wilson , G. Gaul , M. Polsky , J Eng Gas Turbines Power 1996, 565.

[19] S. Bram , J. De Ruyck , Energy Convers Manag 1997.

[20] M. Montero Carrero , W. De Paepe , J. Magnusson , A. Parente , S. Bram , F. Contino , Appl. Therm. Eng. 2017, 796.

[21] C. Wei , S. Zang , Appl. Therm. Eng. 2013, 166.

[22] Y. Wang , Y. Li , S. Weng , M. Su , Energy Convers Manag. 2007, 48, 756.

[23] P. L. Orts‐Gonzalez , P. K. Zachos , G. D. Brighenti , Appl. Therm. Eng. 2018, 545.

[24] Z. Xu , Y. Lu , B. Wang , L. Zhao , C. Chen , Y. Xiao , Energy 2019, 175, 687.

[25] Z. Xu , Y. Xie , Y. Xiao , Appl. Therm. Eng. 2017, 727.

[26] D. Pashchenko , R. Mustafin , I. Karpilov , Appl. Therm. Eng. 2022, 212, 118578.

[27] H. Aygun , O. Turan , Energy 2020, 195, 117008.

[28] H. Aygun , Energy 2022, 245, 123251.

[29] D. Pashchenko , R. Mustafin , I. Karpilov , Energy 2022, 252, 124081.

[30] T. K. Ibrahim , M. M. Rahman , M. K. Mohammed , F. Basrawi , Int J Automot Mech Eng 2016, 13, 3215.

[31] T. K. Ibrahim , M. K. Mohammed , D. W. H. A. Al , A. T. Al‐Sammarraie , F. Basrawi , J Adv Res Fluid Mech Therm Sci 2019, 57, 228.

[32] W. De Paepe , F. Contino , F. Delattin , S. Bram , J. De Ruyck , Appl. Therm. Eng. 2014, 70, 846.

[33] E. Macchi , A. Poggio , Proc ASME Turbo Expo 2015, 4.

[34] N. D. AGren , M. O. Westermark , M. A. Bartlett , T. Lindquist , J Eng Gas Turbines Power 2002, 124, 96.

[35] L. Gillan , V. Maisotsenko , Maisotsenko Open Cycle Used for Gas Turbine Power Generation, Proc. ASME Turbo Expo , ASME, Atlanta, Georgia, USA 2003, pp. 75–84.

[36] A. Omar , M. Saghafifar , M. Gadalla , Appl. Therm. Eng. 2016, 107, 1104.

[37] G. Zhu , T. T. Chow , K. F. Fong , C. K. Lee , Appl. Energy 2019, 113592.

[38] M. Saghafifar , M. Gadalla , Appl. Energy 2015, 149, 338.

[39] R. Tariq , N. A. Sheikh , A. Bassam , J. Xamán , Heat Mass Transfer 2018, 55, 1477.

[40] R. Tariq , N. A. Sheikh , Appl. Therm. Eng. 2018, 133, 49.

[41] W. De Paepe , A. Pappa , M. Montero Carrero , L. Bricteux , F. Contino , Appl. Energy 2020, 279, 115898.

[42] I. Reyzin , Int J Energy a Clean Environ 2011, 12, 129.

[43] H. Caliskan , I. Dincer , A. Hepbasli , Prog. Clean Energy 2015, 1, 41.

[44] M. Saghafifar , M. Gadalla , Appl. Therm. Eng. 2015, 82, 351.

[45] M. Saghafifar , A. Omar , S. Erfanmoghaddam , M. Gadalla , Appl. Therm. Eng. 2017, 111, 431.

[46] M. Saghafifar , M. Gadalla , Appl. Energy 2017, 190, 686.

[47] A. Sohani , Y. Farasati , H. Sayyaadi , Energy Convers Manag 2017, 150, 463.

[48] G. Zhu , T. T. Chow , K. F. Fong , C. K. Lee , X. J. Luo , Energy Convers Manag 2018, 91.

[49] H. Sadighi Dizaji , E. J. Hu , L. Chen , S. Pourhedayat , Energy Convers Manag 2019, 195, 1067.

[50] Z. Guangya , T. T. Chow , K. F. Fong , C. K. Lee , Energy Procedia, 2019.

[51] L. Chen , J. Shen , Y. Ge , Z. Wu , W. Wang , F. Zhu , H. Feng , Energy Convers Manag 2020, 217, 113001.

[52] M. Saghafifar , M. Gadalla , Energy 2015, 87, 663.

[53] A. E. Kabeel , M. Abdelgaied , Energy Convers Manag 2016, 126, 526.

[54] P. Ahmadi , I. Dincer , M. A. Rosen , Energy Convers Manag 2013, 282.

[55] I. Dincer , M. A. Rosen , P. Ahmadi , Optimization of energy systems 2017, 10.1002/9781118894484.

[56] E. Uçkun, [Turkish] Biyodizel yakıtı kullanan bir dizel motorunda ekserji analizi . Available at: http://dspace.kocaeli.edu.tr:8080/xmlui/handle/11493/890. Kocaeli Universitesi, Fen Bilimleri Enstitusu, 2004.

[57] Z. Duan , X. Zhao , J. Li , Energy 2017, 140, 506.

[58] C. Zhan , X. Zhao , S. Smith , S. B. Riffat , Build. Environ. 2011, 46, 657.

[59] L. Wang , C. Zhan , J. Zhang , X. Zhao , Energy 2019, 171, 1206.

[60] H. Caliskan , K. Mori , Energy 2017, 128, 128.

[61] Daily CO2 , CO2‐Earth 2021, https://www.co2.earth/daily‐co2.

[62] I. Dinçer , M. A. Rosen , Exergy: energy, environment, and sustainable development, 2nd ed., Elsevier, 2013.

[63] M. Gürtürk , H. F. Oztop , Appl. Therm. Eng. 2014, 67, 554.

[64] H. Caglayan , H. Caliskan , Appl. Therm. Eng. 2018, 504.

[65] T. J. Kotas , The exergy method of thermal plant analysis, Butterworth‐Heinemann, United States 1985.

[66] J. Szargut , T. Styrylska , (In German) Angenäherte Bestimmung der Exergie von Brennstoffen. Brennstoff‐Wdrme‐Kraft 1964, 16, 589.

[67] J. Szargut , D. Morris , F. Steward , Exergy Analysis of Thermal, Chemical, and Metallurgical Processes: Table of Standard Chemical Exergies, Hemisphere Publishing Corporation, New York 1988.

[68] F. Gharagheizi , P. Ilani‐Kashkouli , R. C. Hedden , Energy 2018, 158, 924.

[69] M. J. Moran , H. N. Shapiro , D. D. Boettner , M. B. Bailey , Fundamentals of Engineering Thermodynamics, 8th ed., Wiley, 2014.

[70] B. Adrian , T. George , M. Michael , Thermal design & optimization, John Wiley Son, Inc., New York 1996.

[71] I. S. Ertesvåg , Energy Convers Manag 2007, 48, 1983.

[72] R. Tariq , N. A. Sheikh , J. Xamán , A. Bassam , Appl. Energy 2018, 228, 789.

[73] R. Kalbasi , F. Izadi , P. Talebizadehsardari , J. Therm. Anal. Calorim. 2020, 2913.

[74] H. Caliskan , A. Hepbasli , I. Dincer , V. Maisotsenko , Int J Refrig 2011, 34, 980.

[75] M. Shukuya , A. Hammache , VTT Tiedotteita – Research Notes T2158, Espoo, Finland: 2002.

[76] H. Caliskan , I. Dincer , A. Hepbasli , Energy Convers Manag 2012, 56, 69.

[77] Software F‐Chart , EES: Engineering Equation Solver: Engineering Software. F‐Chart Softw 2015, 2012, 6.

[78] E. W. Lemmon , R. T. Jacobsen , S. G. Penoncello , D. G. Friend , J. Phys. Chem. Ref. Data 2000, 29, 331.

[79] H. Keenan , J. Chao , J. Kaye , Gas tables: International version second edition (SI units), Vol. 30, John Wiley & Sons, 1983.

[80] Y. S. Touloukian , S. C. Saxena , P. Hestermans , Thermophysical properties of matter – the TPRC data series 1975, 11.

[81] Y. S. Touloukian , P. E. Liley , S. C. Saxena , Thermal Conductivity – Nonmetallic Liquids and Gases, Vol. 3, Defense Technical Information Center, 1970.

[82] B. J. McBride , M. J. Zehe , G. S. N. Glenn , Coefficients for Calculating Thermodynamic Properties of Individual Species 2002.

[83] N. B. Vargaftik , Handbook of physical properties of liquids and gases : pure substances and mixtures, 2nd ed., Springer‐Verlag, Berlin Heidelberg Germany 1975.

[84] R. W. Hyland , A. Wexler , Formulations for the Thermodynamic Properties of the Saturated Phases of H2O from 173.15 K to 473.15 K, 1983.

[85] E. Van den Bulck , Analysis of Solid Desiccant Rotary Dehumidifiers, University of Wisconsin, 1983.

[86] E. Van den Bulck , Convective Heat and Mass Transfer in Compact Regenerative Dehumidifiers, University of Wisconsin, 1987.

[87] The Language of Technical Computing 2019. https://www.mathworks.com/products/matlab.html

[88] STATGRAPHICS Centurion 2021. https://www.statgraphics.com/ (accessed May 11, 2021).

[89] R. J. Neufeld , Heating values of hydrogen and fuels, reading material for CHEE332 Design Project course, Department of Chemical Engineering, Faculty of Engineering and Applied Science, Queen's University, Ontario, Canada.

[90] SIEMENS , SGT‐800 Industrial Gas Turbine, Germany 2009.

[91] M. Saghafifar , Thermo‐Economic Optimization of Hybrid Combined Power Cycles Using Heliostat Solar Field, Masters Degree Thesis, Submitted at College of Engineering American University of Sharjah, 2016.

[92] B. Riangvilaikul , S. Kumar , Energy Build. 2010, 42, 2241.

[93] B. Riangvilaikul , S. Kumar , Energy Build. 2010, 42, 2241.

[94] R. Tariq , N. A. Sheikh , J. Xamán , A. Bassam , Build. Environ. 2019, 152, 105.

[95] F. Hermann , J. Klingmann , R. Gabrielsson , Am. Soc. Mech. Eng. Int. Gas Turbine Institute 2003, Turbo Expo IGTI, 1, American Society of Mechanical Engineers Digital Collection, pp. 819–827.

